# Incidence, Prevalence, and Outcomes of Pediatric Trauma in Rural Appalachia (West Virginia) From 2017 to 2019

**DOI:** 10.7759/cureus.14245

**Published:** 2021-04-01

**Authors:** Pavithra Ellison, Daniel Cifarelli, Alexandra Pearce, Lucas Moore, Dan Parrish, Matthew Ellison, Alyssa Fazi, Trey Vanek, Hal Meltzer, Jennifer Knight

**Affiliations:** 1 Anesthesiology, West Virginia University School of Medicine, Morgantown, USA; 2 Neurosurgery, West Virginia University School of Medicine, Morgantown, USA; 3 Medicine, West Virginia University School of Medicine, Morgantown, USA; 4 Biostatistics and Epidemiology, West Virginia University School of Medicine, Morgantown, USA; 5 Surgery, West Virginia University School of Medicine, Morgantown, USA; 6 Surgical Trauma and Critical Care, West Virginia University School of Medicine, Morgantown, USA

**Keywords:** trauma pediatric, child abuse, toxicology screen, patient outcomes

## Abstract

Background

Appalachian rural pediatric trauma has its unique incidence, presentation, and distribution due to the mechanisms of injury, geographic location, access to care, and social issues.

Purpose

To review, analyze, and understand pediatric trauma in West Virginia during the period 2017-2019.

Materials and methods

After institutional review board approval, the statewide trauma database was queried and analyzed in a retrospective cohort study for all pediatric trauma ages zero to 18 from 2017-2019 in the Appalachian regions one through four in West Virginia.

The following were analyzed: gender, injury mechanism, Glasgow Coma Scale Score (GCS) at admission, injury severity score (ISS), toxicology screen results, hospital length of stay, duration of ventilatory support, number of procedures performed during admission, presence of non-accidental trauma, cardiac arrest, patient discharge disposition, and mortality.

Results

One-thousand eighty-two (1182) patients between the ages of zero to 18 were admitted to the trauma center. An average of 37% was female and 63% male. In the 11-18 age group, 24% were female and 76% were male. Most injuries were due to blunt force (89%), followed by penetrating injuries (7.2%) and burns (1.4%). The majority had minor or moderate injuries with 95% receiving a Glasgow Coma Scale (GCS) >13 and 72% listed as minor on the injury severity score (ISS). Children in ages 0-2 years had the highest proportion of poor (0-8) GCS scores, high ISS (>14) scores, most hospital admission days, most days on a ventilator, highest mortality, most pre-hospital cardiac arrests, child abuse, burns, and placement with child protective services. An average of 31% of children tested, and 17% in the age group of 0-2 had a positive toxicology screen. There were 3670 procedures done in total and the most common procedure performed was an ultrasound of the abdomen. Procedures were performed in 90% of the patients.

Conclusions and relevance

Based on our study, the zero to two-year-old pediatric trauma patients are most vulnerable to poor outcomes and may need targeted preventative interventions. Toxicology screens may need to be more widely implemented in pediatric trauma in the Appalachian region.

Rural trauma in Appalachia has endemic issues related to substance abuse, poverty, and a lower degree of social support as compared to urban areas. Although the distribution of injury may follow a national distribution, mechanism, management, and outcomes can vary.

## Introduction

For the past three decades, there has been an evolving trend towards regional centers of excellence for specialty care in pediatric cancer, cardiovascular medicine, and solid organ transplantation [1-4]. However, the ability of patients and their families to choose a specialty center is unfeasible for urgent traumatic injury, leaving local pediatric trauma designated facilities as the first and only option for management [5]. Maintaining practice standards with a routine review of patient outcomes in regional trauma centers provides metrics for outcomes improvement.

Rural Appalachian pediatric trauma centers encounter patients with unique injury mechanisms and whose demographics are dissimilar from those in urban centers. Therefore, outcomes data from urban or suburban centers may or may not be relevant in the rural setting [6]. In this retrospective cohort study, we reviewed the admission and disposition data of pediatric trauma patients at our institution (the largest pediatric trauma center in the state) over a three-year period. This facility serves as the primary referral center for a rural population, with a density of approximately 77 people per square mile. When compared to the adjacent states of Pennsylvania and Ohio, with 286 people per square mile, the data reviewed in this study could provide a better means of providing optimal care and, ultimately, injury prevention in a rural population.

## Materials and methods

Methods

We conducted a retrospective cohort study in which we aimed to describe the incidence or prevalence of different pediatric trauma in rural Appalachia and describe its association with variables of interest, including gender, injury mechanism, Glasgow Coma Scale Score (GCS) at admission, injury severity score (ISS), drug test results, hospital length of stay, duration of ventilatory support, number of procedures performed during admission, presence of non-accidental trauma, cardiac arrest, patient discharge disposition, and mortality. After approval from our institutional review board (IRB), the admission and outcomes database maintained by our health system was queried for all admissions between 2017 and 2019. The primary inclusion criterion was an age of less than 18 years at the time of presentation.

Statistics

Statistical analysis was performed using the Statistical Package for the Social Sciences (SPSS) software v. 26 (IBM Corporation, Armonk, NY). The data were stratified based on age, with primary groupings of zero to two years, three to five years, six to 10 years, and 11-18 years, and used for the determination of mean, percentages, and interquartile range. The minimum variables obtained included gender, injury mechanism, GCS at admission, ISS, toxicology screen results, hospital length of stay, duration of ventilatory support, number of procedures performed during admission, presence of non-accidental trauma, cardiac arrest, patient discharge disposition, and mortality.

## Results

Patient demographics

During the period of 2017-2019, a total of 1,182 pediatric trauma patients were admitted to health system facilities for treatment. Of these, 739 (63%) were identified as male. As patient age increased, there was a predominance of trauma in males, reaching a maximum of 76% male patients in the 11 to 18-year-old patient cohort (summarized in Table 1).

**Table 1:** Pediatric trauma categorization by gender

Age (years)	Female n (%)	Male n (%)	Unknown n (%)
0 - 2	125 (44.17)	158 (55.83)	0 (0.00)
3 - 5	108 (44.63)	133 (54.96)	1 (0.41)
6 - 10	136 (38.64)	216 (61.36)	0 (0.00)
11 - 18	72 (23.61)	232 (76.07)	1 (0.33)
Total	441 (37.31)	739 (62.52)	2 (0.17)

Regional pediatric trauma distribution

Based on districts identified by the West Virginia Department of Health and Human Resources Bureau for Children and Families, the state is divided into four regions, Regions 1-4.

Most patients in this study were from Regions 1 and 3 (n=1028). Sixty-nine percent or 581 out of 842 patients from Region 1 were directly admitted to the largest tertiary institution. In this study, 101 patients were referred from facilities outside of West Virginia, including 72 from Maryland, eight from Ohio, 19 from Pennsylvania, and two from Virginia (Table 2).

**Table 2:** Pediatric trauma categorization by age and region

Age (years)	Region 1 n (%)	Region 2 n (%)	Region 3 n (%)	Region 4 n (%)
0 - 2	194 (76.68)	3 (1.19)	46 (18.18)	10 (3.95)
3 - 5	181 (81.90)	2 (0.90)	34 (15.38)	4 (1.81)
6 - 10	252 (77.06)	4 (1.22)	59 (18.04)	12 (3.67)
11 - 18	215 (76.79)	4 (1.43)	47 (16.79)	14 (5.00)
Total	842 (77.89)	13 (1.20)	186 (17.21)	40 (3.70)

Presentation of pediatric trauma injuries

Patients were classified into the three major injury categories, which include blunt trauma (including motor vehicle collision), burns, and penetrating injury (including gunshot wounds). Most injuries were caused by blunt trauma (89% across all age groups), with burns and penetrating injuries comprising 1.4% and 7.2%, respectively. A trend towards a higher incidence of burns, relative to other injury mechanisms, was seen in patients aged zero to two years (76%), as compared to 7%-11% in all other age cohorts, while the incidence of penetrating injuries was evenly distributing based on age (Table 3).

**Table 3:** Pediatric trauma categorization by mechanism of injury N/A: not documented

Age (years)	Blunt n (%)	Burn n (%)	Penetrating n (%)	Other n (%)	N/A n (%)
0 - 2	243 (85.87)	13 (4.59)	19 (6.71)	7(2.47)	1 (0.35)
3 - 5	216 (89.26)	1 (0.41)	16 (6.61)	6 (2.48)	3 (1.24)
6 - 10	316 (89.77)	1 (0.28)	28 (7.95)	3 (0.85)	4 (1.14)
11 - 18	277 (90.82)	2 (0.66)	23 (7.54)	1(0.33)	2 (0.66)
Total	1052 (89.00)	17 (1.44)	86 (7.28)	17(1.44)	10(0.85)

Level of consciousness and injury severity at admission

Most patients had minor or moderate injuries based on GCS and ISS (95% had GCS > 13 and 72% were listed as minor on the ISS). For severe injuries, patients in the zero to two-year age group had the highest incidence of poor GCS and ISS scores (53% of patients with GCS ≤8 and 56% of patients designated as severe on the ISS (≤48) fell in this age group (summarized in Tables 4-5).

**Table 4:** Pediatric trauma categorization by GCS score at time of admission GCS: Glasgow Coma Score

Age (years)	Severe (0-8) n (%)	Moderate (9-12) n (%)	Mild (13-15) n (%)	Unknown n (%)
0 - 2	17 (6.01)	5 (1.77)	260 (91.87)	1 (0.35)
3 - 5	5 (2.07)	4 (1.65)	233 (96.28)	0 (0.00)
6 - 10	6 (1.70)	7 (1.99)	339 (96.31)	0 (0.00)
11 - 18	4 (1.31)	3(0.66)	299 (98.03)	0 (0.00)
Total	32(2.71)	18(1.52)	1131 (95.69)	1 (0.08)

**Table 5:** Pediatric trauma categorization by ISS at time of admission ISS: injury severity score

Age (years)	Minor (%)	Moderate (%)	Serious (%)	Severe (%)	Critical (%)
0 - 2	188 (66.43)	69 (24.38)	11 (3.89)	14 (4.95)	1 (0.35)
3 - 5	179 (73.97)	56 (23.14)	3 (1.24)	4 (1.65)	0 (0.00)
6 - 10	276 (78.41)	57 (16.19)	12 (3.41)	7 (1.99)	0 (0.00)
11 - 18	212 (69.51)	78 (25.57)	9 (2.95)	5 (1.64)	1 (0.33)
Total	855 (72.34)	260 (22.00)	35 (2.96)	30 (2.54)	2 (0.17)

Toxicology screen

A total of 275 patients (23%) underwent urine or serum toxicology screen in the emergency department. Of these, 85 (31%) were found to be positive. There was an increasing percentage of positive toxicology screens with advancing patient age as seen in the following groups (Age 0-2: 18% (7/39), 3-5: 21% (12/57), 6-10: 35% (29/82), 11-18: 37/97 (38%)). Overall, 7.19% of children in the study had a positive toxicology screen (summarized in Table 6).

**Table 6:** Pediatric trauma toxicology testing and results NTDB: National Trauma Data Bank®

Age (years)		Testing completed n (%)	Positive n (%)		No Testing n (%)		
0 - 2		39 (13.78)	7 (17.95)		244 (86.22)		
3 - 5		57 (23.55)	12 (21.05)		185 (76.45)		
6 - 10		82 (23.30)	29 (35.37)		269 (76.42)		
11 - 18		97 (31.80)	37 (38.14)		208 (68.20)		
Total		275 (23.27)	85 (30.91)		906 (76.65)		
National Averages for Alcohol and Drug Use in Pediatric Trauma Cases in 2016 Source: NTDB Pediatric Report 2016. Published online 2016:128							
Alcohol use	Number (%)			Drug use		Number (%)	
No (confirmed by test)	25109 (17.80)			No (confirmed by test)		9661 (6.85)	
No (not tested)	88850 (62.99)			No (not tested)		103184 (73.15)	
Yes (beyond the legal limit)	2953 (2.09)			Yes (confirmed by test; illegal drug use)		6031 (4.28)	
Yes (trace amounts)	2066 (1.46)			Yes (confirmed by test; prescription drug)		3519 (2.49)	
Not Applicable	16725 (1.86)			Not Applicable		10574 (7.50)	
Not Known/Not Recognized	5348 (3.79)			Not Known/Not Recognized		8082 (5.73)	
Total	141051 (100)			Total		141051 (100)	

Children between the ages of zero to two had the highest proportion of poor GCS scores (0-8). High ISS scores (> 14), most hospital admission days, most days on a ventilator, highest mortality, most pre-hospital cardiac arrests, the highest incidence of child abuse, most burns, and highest placement with child protective services.

Hospital course and discharge outcomes

Patients aged zero to two had the longest length of stay, most days on a ventilator, highest mortality, most prehospital cardiac arrests, and the highest incidence of child abuse. The average length of stay for patients in all age groups was 2.61 ± 4.67 days (Tables 7-8).

**Table 7:** Pediatric trauma categorization by age for length of stay in hospital (days) *STDEV: standard deviation

Age (years)	Number in age group	Mean (STDEV*)	Minimum, Maximum
0 - 2	283	3.42 (8.22)	0,90
3 - 5	242	2.19 (1.60)	0,16
6 - 10	352	2.34 (2.99)	0,34
11 - 18	305	2.48 (2.92)	0,37

**Table 8:** Pediatric trauma categorization by age for assisted ventilation (days) STDEV: standard deviation

Age (years)	Number	Mean (STDEV*)	Minimum, Maximum
0 - 2	23	10.30 (14.18)	1, 61
3 - 5	9	2.55 (1.24)	1,5
6 - 10	11	3.54 (3.78)	1,12
11 - 18	8	2.62 (1.84)	1,7

Overall, 15 patients (1.27%) did not survive their injuries. Ten of these belonged to the zero to two age group (3.53%), one in the three to five age group (0.41%), one in the six to 10 age group (0.28%), and three in the 11-18-year-old age group (0.98%). All patients, except those in the zero to two-year-old age group, had less than 1% mortality (Table 9).

**Table 9:** Pediatric trauma categorization by age and condition at discharge

Age (years)	Alive n (%)	Deceased n (%)
0 - 2	273 (96.47)	10 (3.53)
3 - 5	241 (99.59)	1 (0.41)
6 - 10	351 (99.72)	1 (0.28)
11 - 18	302 (99.02)	3 (0.98)
Total	1167 (98.73)	15 (1.27)

The incidence of prehospital cardiac arrest in all patients was 1.18% (n=14). It was less than 1% in all age groups except those aged zero to two, where it was 3.18% (n=9) (Table 10).

**Table 10:** Pediatric trauma categorization by age and by pre-hospital cardiac arrest Yes: History of pre-hospital cardiac arrest No: No history of pre-hospital cardiac arrest

Age (years)	Yes Number (%)	No Number (%)
0 - 2	9 (3.18)	274 (96.82)
3 - 5	0 (0.00)	242 (100)
6 - 10	3 (0.85)	349 (99.15)
11 - 18	2 (0.66)	303 (99.34)
Total	14 (1.18)	1168 (98.82)

Similarly, the overall incidence of child abuse was 1.52% (n=18), with the overwhelming majority in the zero to two-year-old age group (n=15). The age group with the second-highest occurrence was the three to five-year-old group (n=2), followed by the six to 10 age category (n=1). There were no reported instances of child abuse in the 11-18 category (Table 11).

**Table 11:** Pediatric trauma categorization by age and incidence of child abuse

Age (years)	Number (%)
0 - 2	15 (5.30)
3 - 5	2 (0.83)
6 - 10	1 (0.28)
11 - 18	0 (0.00)
Total	18 (1.52)

The age group with the highest incidence of child protective services (CPS) involvement or burn center referral was zero to two years at 12%.

There was a total of 3670 procedures performed in all patients, the most common of which was the abdominal ultrasound. Procedures were performed in 1063 (90%) of all patients. The average number and standard deviation of procedures performed in each patient were approximately 3.4 ± 3 (Table 12).

**Table 12:** Pediatric trauma categorization by age and procedures performed STDEV: standard deviation

Age Category (years)	Number (%)	Mean (STDEV)	Minimum, Maximum
0 - 2	224 (79.15)	3.34 (3.77)	1, 26
3 - 5	221 (91.32)	2.82 (2.04)	1,14
6 - 10	335 (95.17)	3.42 (2.82)	1,29
11 - 18	283 (92.79)	4.05 (3.34)	1,26

The percentage of patients who underwent at least one procedure in each group were: 79% in the zero to two age group (3.34 ± 3.77 procedures with a maximum number of 26); 91% in the three to five-year-old age group (2.82 ± 2.04 procedures with a maximum of 14); 91% in the six to 10 age group (3.42 ± 2.82 procedures with a maximum of 29); 95% in the 11-18-year-old age group (3.42 ± 2.82 procedures with a maximum of 29).

## Discussion

Unintentional trauma is the number one cause of death in children and adolescents in the United States [7]. Although pediatric trauma populations can be similarly characterized, there are unique challenges faced in different regions of the country. In this investigation, we analyzed the characteristics of pediatric trauma patients served by the largest tertiary hospital and our state health system. Upon review of the literature, it is evident that there are differences between urban and rural pediatric trauma populations [5-6].

A common theme throughout the literature is that males have a higher propensity for traumatic injury than females, as well as a higher incidence of death due to traumatic injury [8-11]. Our data demonstrated a comparable trend, with 63% of all analyzed pediatric trauma cases being male. We also found that there was an increase in the proportion of males versus females experiencing traumatic injuries with rising patient age. This likely reflects the increased risk-taking behavior seen in adolescent males relative to other age and gender categories. Accordingly, a similar study in Illinois found that higher age and male gender were strong predictors for a positive toxicology screen among their pediatric trauma cases [12]. Additional data are needed to determine if this is true for our population, but it is important to consider the relationship between drug use, male gender, and age and how it could contribute to the trend seen in our study population.

Drug use in pediatric patients has not only been associated with increasing age and male gender but also with lower socioeconomic status and more violent injury mechanism [13]. In our study population, 23% of all patients were screened for drug use. Thirty-one percent of these tests were positive, with 38% of the positive tests in the 11-18-year-old age group. Several other studies have demonstrated a similar proportion of positive drug screens [11-14]. One study demonstrated positive tests in 39% of the 12-18-year-old trauma group who met the criteria for drug screening while another revealed a 34% positive rate in 13-19-year-old trauma patients [13-14]. However, this is not ubiquitous across the literature. For example, Indiana and Illinois have significantly different incidences of positive screens; 18% versus 9% in their adolescent trauma population [10-11]. Based on records from the 2016 National Trauma Data Bank, 4.28% of pediatric trauma cases tested positive for illegal drugs and 2.09% tested positive for alcohol content above the legal limit [15]. Although our data does not differentiate between drug and alcohol use, 7.2% of our pediatric study population had a positive drug test, suggesting that the Appalachian region may have more drug-positive pediatric trauma cases than the national average. Despite variances in literature, the proportion of pediatric trauma cases with positive toxicology screens in our region is in the upper limit of reported data. Further studies to ascertain whether pediatric trauma, its mechanism of injury, associated factors like drug and alcohol association have evolved and changed over the last decade in the Appalachian region. Our goal in the next study is to analyze the evolution of trauma and its management and outcomes over the last decade in the Appalachian region. An in-depth analysis of the subtypes of the toxicology screen, correlating it to injury type (violent versus non-violent injuries, accidental versus intentional injuries), age and sex, and race may give us an opportunity for early preventative intervention. One of the shortcomings of this pilot study was the lack of trauma distribution based on race. Future studies will include race in their analysis.

Although the classification of injury mechanisms may differ in the literature, one common theme is the high prevalence of blunt force trauma-related injuries in pediatric populations [8,10]. Our data follow a similar pattern, with 89% of all reported injuries secondary to blunt force trauma. It is important to note that motor vehicle accidents are included in this category. The World Health Organization reported that road-related injuries are the leading cause of death in children aged 15-19 and the second leading cause in those aged five to 14 in the United States [16]. Another concerning point is the high incidence (5%) of child abuse in the zero-to-two-year-old age group that significantly exceeds that seen in any other age group. This clearly demonstrates a need for hypervigilance in an extremely vulnerable group of patients. The zero-to-two-year-old group also had injuries that were more severe, as well as the highest proportion of the following: poor (0-8) GCS scores, high ISS (>14) scores, longer length of stay, increased ventilator duration, higher mortality, more pre-hospital cardiac arrests, more burns, and higher incidence of placement with CPS. This suggests that our injury prevention strategies may need to specifically target parents with children at or under the age of two.

In the future, as we expand the cohort to the last decade of data, we hope to utilize the information gathered to help guide the development of prevention strategies targeting the most common injuries seen in rural pediatric populations.

## Conclusions

Male patients represented almost 2/3 of pediatric trauma with blunt force injuries being the most predominant mechanism of injury. Zero-to-two-year-old patients were the most vulnerable to poor outcomes and may need targeted preventative interventions. Due to the high incidence of positive toxicology screens, these tests may need to be more widely implemented in pediatric trauma in the Appalachian region. Overall, there are many similarities between the information that we have ascertained from rural pediatric trauma from the Appalachian region and trauma records across the United States. However, there were also many parameters, namely, the high incidence of positive toxicology screens, which may uniquely reflect the population we serve. Rural pediatric trauma may have endemic issues related to substance abuse, poverty, and lower degrees of social support when compared to urban areas. Nevertheless, substantial work is needed to delineate specific trends in various parameters like injury mechanisms across age groups. This will be an important aid to the development of prevention strategies to target our most vulnerable population: the children of the Appalachian region.
